# Steam explosion processing intensifies the nutritional values of most crop byproducts: Morphological structure, carbohydrate-protein fractions, and rumen fermentation profile

**DOI:** 10.3389/fnut.2022.979609

**Published:** 2022-10-17

**Authors:** Liwen He, Yinchao Huang, Lei Shi, Zhenming Zhou, Hao Wu

**Affiliations:** State Key Laboratory of Animal Nutrition, College of Animal Science and Technology, China Agricultural University, Beijing, China

**Keywords:** chemical composition, crop byproduct, nutritional value, rumen fermentation, steam explosion

## Abstract

To investigate the feasibility of steam explosion on the exploitation of ruminant feedstuff, the morphological structure, carbohydrate-protein fractions, and rumen fermentation profile of five typical crop byproducts (corn cob, rice straw, peanut shell, millet stalk, and sugarcane tip) were analyzed before and after steam explosion processing. The results showed that these crop byproducts had different physicochemical properties and rumen fermentation profiles, most of which could be improved by steam explosion processing, i.e., more rough morphological surface, much-broken structure, more digestible carbohydrate fraction (non-NDF +49.92–452.24%), faster gas production rate (c +9.72–68.75%), higher dry matter digestibility (DMD_48_ +11.38–47.36%), more available energy (ME −3.69–+42.13%, except for peanut shell), along with more unavailable protein fraction (ADICP +27.16–102.70%). It is suggested that steam explosion processing could intensify the feeding value of most crop byproducts for ruminants, but with a caution of heat damage to proteins.

## Introduction

With the explosion of the global population and expense demand, the challenges of food supply and other requirements are increasingly conspicuous across the world. Exploiting unconventional feedstuff has been becoming a common method to combat the shortage and high expense of energy and feed supply in the industries of husbandry (1). Crop byproduct, such as stover/straw, cob, hull/shell, or bagasse, is one kind of the most common non-food feedstuff, with the characteristics of low cost, huge quantity, and various origins, and its annual yield globally is roughly estimated to be 3 billion tons^1^. It could be considered a great resource or source of problems because such byproducts are badly in need of proper disposal to maintain a sustainable production system. The utilization of crop residues would avoid open burning, accelerate the rotation of crops, and increase production efficiency on a limited land field (2, 3). Making full use of such resources would be of great benefit to the increase of income and sustainable development of the livestock industry. However, raw crop byproducts are usually low in nutrient density and high in lignocellulose content with tough recalcitrance structure, leaving them with poor nutritional value for animals. To be efficiently used, proper pretreatment is needed to remove the hindrance of enzymatic hydrolysis or microorganism utilization (4).

Many attempts have been made to intensify the utilization of such abundant feed resources. Among the alternatives, steam explosion processing sounds like a promising physicochemical pretreatment that effectively breaks up the lignocellulosic structure and alters hemicellulose and lignin contents by the effects of steam cooking on the duration and steam shear force at explosion (5, 6). It has become a common pretreatment method to destroy biomass recalcitrance in biofuel and pulping production. Plenty of studies show that steam explosion processing results in dramatical structure changes, lower cellulose degree of polymerization, higher lignocellulose porosity, and more efficient saccharification of enzymatic hydrolysis (7–9). Meanwhile, the rumen is a natural and highly specialized fiber degradation system, where the plant cell wall is efficiently deconstructed by resident microorganisms. Morphological structure and nutrient content of feedstuff, as well as rumen microorganism flora, are critical factors dictating digestion. It is believed that such alteration in the biomass during the process of the steam explosion would likely contribute to the fermentation of rumen microorganisms. Much practice proves that multiple combinations of physical methods, chemical methods, and biological methods would achieve a satisfactory processing result relative to a single method (7, 10, 11). Thus, it is hypothesized that the combination of steam explosion processing and rumen fermentation would promote the utilization of crop byproducts, i.e., steam explosion processing would improve the feeding value when crop byproducts are to feed ruminants.

In the present study, five typical crop byproducts (corn cob, rice straw, peanut shell, millet stalk, and sugarcane tip) were processed by the steam explosion under specific conditions, mainly paying attention to the changes in the morphological structure, carbohydrate and protein fractions, rumen fermentation profile, and effective energy for beef cattle, to investigate the feasibility of steam explosion processing on the exploitation of such crop byproducts for ruminant feeding.

## Materials and methods

### Raw material preparation and steam explosion processing

Five regional crop byproducts, i.e., corn cob, rice straw, peanut shell, millet stalk, and sugarcane tip, were collected from their major producing areas, cut into 2–5 cm lengths with a straw breaker, and kept in air-dry conditions until used. Based on the results (texture, dry matter, and color) of the preliminary test, the processing conditions (pressure and residence time) of steam explosion were set as 1.0 MPa+180 s, 1.7 MPa+60 s, 1.1 MPa+300 s, 1.5 MPa+90 s, and 1.5 MPa+30 s for corn cob, rice straw, peanut shell, millet stalk, and sugarcane tip, respectively. Steam explosion processing was performed in a pilot scale steam explosion equipment (Zhengdao Environmental Protection Technology Co., Ltd, Henan, China), whose reactor volume was 50 L and maximum working pressure was 3.0 MPa. Before and after steam explosion, five kinds of materials were individually sampled, and all the processing treatments were conducted in triplicate. All the samples were destined for analyses of morphological structure observation, chemical composition, and rumen fermentation.

### Structural observation and functional group analysis

The appearance of raw materials before and after the steam explosion processing was recorded using a digital camera (FUJI X-T20, Japan) and then scanned on a scanning electron microscopy (SEM; SU-3500, Japan) at an appropriate magnification. Prior to imaging, representative samples were fixed on a scanning plate and sputter-coated with platinum (ALTO1000 gold plating instrument) to make them conductive, and then kept in a drying closet until analysis.

The Fourier-transform infrared **(FTIR**) spectra of feedstuff at 4,000–400 cm^−1^ before and after processing were scanned by using an FTIR spectrophotometer (Bruker Vertex 70, Bruker, Ettlingen, Germany) equipped with an RT-DLaTGS Detector with a resolution of 4 cm^−1^ and 1 scan per sample. Referring to the sample preparation, the dry sample was finely ground using a small pulverizer equipped with a 160-mesh screen and then manually ground and mixed with pure KBr (1.5 mg+100 mg) by agate mortar, and finally, the powdery mixture was pressed into a tablet using molding equipment. Before data collection, background scanning was performed for baseline correction.

Meanwhile, X-ray diffraction (**XRD**) was recorded using a diffractometer (Siemens D-5000, Bruker, Ettlingen, Germany), where Cu/Kα radiation was generated at 40 kV and 40 mA. Each sample was scanned in the range of 3–70° with a step size of 0.02 and 0.3 s per step. The cellulose crystallinity index (**CrI**) was estimated using the following formula (12):


$$\begin{array}{l} \text{CrI }=\;\left({\text{I}}_{002}\;-\;{\text{I}}_{\text{am}}\right)/{\text{I}}_{002} \end{array}$$

where I_002_ is the scattered intensity at the main peak (2θ = 22.5°), and I_am_ is the scattered intensity due to the amorphous portion evaluated as the minimum intensity (2θ = 18°) between the main and secondary peaks.

### Chemical composition analysis

All the samples were dried in a 65°C oven and then milled in a pulverizer equipped with a 1 mm sieve. Carbohydrate and protein fractions were fully analyzed and calculated referring to the methods of the Cornell Net Carbohydrate-Protein System (CNCPS). In detail, neutral detergent fiber (**NDF**), acid detergent fiber (**ADF**), and acid detergent lignin (**ADL**) were analyzed by Fiber Analyzer (A220, ANKOM Technology Corp., USA) as Van Soest et al. (13), where the content of hemicellulose and cellulose was calculated by the fractional differences of NDF, ADF, and ADL. Water soluble carbohydrates (**WSCs**) were determined by the colorimetric method with 3,5-dinitrosalicylic acid (14). The starch content was measured by a colorimetric method in accordance with the instructions provided in the test kits (Megazyme, Ireland). For the analysis of free monosaccharides and disaccharides, the feed sample was suspended in 0.1 N sodium carbonate by ultrasound extraction, and then the supernatant was screened with a 45 μm filter. The prepared samples were analyzed on a Dionex ion chromatograph (ICS 3000, Dionex, USA) equipped with ED 3000 ampere detector and CarboPac PA10 column (250 mm × 4 mm), running with 250 mmol/L sodium hydroxide as a leachate liquid. Individuals were identified by the retention time referring to the standards and quantified by the peak area.

Crude protein (CP), neutral detergent insoluble crude protein (NDICP), and acid detergent insoluble crude protein (ADICP) were analyzed by the Dumas combustion method using an automatic nitrogen analyzer (Rapid N III, Elementary, Germany). Nonprotein nitrogen (NPN), true protein (TP), and soluble crude protein (SCP) were determined according to the method of Licitra et al. (15). Additionally, ether extract (EE) was measured using an automatic Soxhlet extractor (Ankom XT15i, Ankom Technology, USA), and crude ash was measured by calcination in a 550°C Muffle furnace for 5 h.

### Rumen fermentation analysis

The rumen fermentation profile of the materials before and after steam explosion processing was evaluated by *in vitro* rumen culture (gas production and digestibility). *In vitro* incubation was carried out according to the procedure of Menke et al. (16). Before the morning feeding, fresh rumen fluid was collected from three rumen fistulated Angus steers that were reared by 1.3× maintenance nutrient requirement and fed two times per day.

For *in vitro* gas production trial, incubation fluid was made up by mixing fresh rumen fluid with a buffer solution in a ratio of 1:2 (v/v). Each sample of 0.22 g (on an air-dry basis) was weighed into a 100 ml glass syringe in six replicates, and a volume of 30 ml incubation fluid was dispensed into each syringe and then incubated in a shaking bath at 39°C and 60 rpm/s after air was expelled. The volume of gas production (**GP**) was recorded manually at the setting time points of 0, 2, 4, 6, 8, 10, 12, 16, 20, 24, 30, 36, 42, 48, 54, 60, 72, and 96 h of incubation. Subsequently, the reactions in the three syringes containing each sample were terminated after 24 h of incubation, and then fermentation fluid was collected to measure the pH, volatile fatty acids (**VFAs**), and ammonia-nitrogen (**NH**_**3**_**-N**). The pH value was directly measured using a portable pH meter (PHS-3C, Shanghai Leizi Instrument Factory, Shanghai, China), and NH_3_-N concentration was determined according to the method of Broderick and Kang (17). The VFA profile was analyzed with GC 3420 gas chromatograph fitted with HP-INNO wax capillary column (30 m × 0.32 mm) according to the method stated by Erwin et al. (18).

Meanwhile, *in vitro* rumen digestibility was carried out with nylon bags and incubation tubes based on the method of Tilley and Terry (19). The incubation fluid was made up by mixing fresh rumen fluid with buffer solution in a ratio of 1:1 (v/v). Each sample of 0.5 g was weighted into a special nylon bag (8 cm by 3.5 cm, 300 mesh) in eight replicates, which were randomly paired in an incubation tube (100 ml) capped with a one-way gas valve. The content of each tube was added to 80 ml of incubation fluid and then incubated in a shaking bath as aforementioned. After 24 h and 48 h of incubation, two tubes of each sample were randomly terminated, and then the nylon bags were cleaned with deionized water until the wash water was clear, and finally dried at 65°C for 48 h to calculate the digestibility of dry matter (**DMD**).

### Calculation equation

Cumulative gas production records of each syringe were fitted with the following model using the non-linear option of the Statistical Analysis System (SAS 9.0, SAS Institute Inc., Cary, NC, USA):


$$\begin{array}{l} \text{GP }=\text{ b }\times \;\left(1-{\text{e}}^{-\text{ct}}\right) \end{array}$$

where GP (ml/0.2 g DM) is the volume of cumulative GP at time t, b (ml/0.2 g DM) is the asymptotic GP, and c (/h) is the rate of gas production. The effective energy content of the feed was calculated according to Menke and Steingass (20) and NASEM (21):


$$\begin{array}{l} \text{ ME }(\text{MJ/kg})\text{ = 2}.\text{20 + 0}.\text{1357 }\times \;GP\text{ + 0}.\text{0057 }\times \;CP\\ \text{ + 0}.\text{0002859 }\times \;CP^{2}\\ {\text{NE}}_{\text{m}}\;(\text{Mcal/kg})\text{ = 1}.\text{37 }\times \;ME\text{ - 0}.\text{138 }\times \;ME^{2}\text{ + 0}.\text{0105}\\ \;\times \;ME^{3}\text{ - 1}.\text{12}\\ {\text{ NE}}_{\text{g}}\;(\text{Mcal/kg})\text{ = 1}.\text{42 }\times \;ME\text{ - 0}.\text{174 }\times \;ME^{2}\text{ + 0}.\text{0122}\\ \;\times \;ME^{3}\text{ - 1}.\text{65} \end{array}$$

where ME denotes metabolizable energy, and NEm and NEg denote net energy for maintenance and growth, respectively, GP is the cumulative gas production of 0.2 g DM sample after 24 h of incubation, and CP is the crude protein content presented in g/kg DM.

Based on their chemistry and functionality, carbohydrates can be broadly partitioned into non-neutral detergent fiber (**non-NDF**) carbohydrates and neutral detergent fiber (**NDF**) carbohydrates, where non-NDF = 100-(CP+NDF+Fat+Ash). In the newly revised Nutrient Requirements of Beef Cattle (21), dietary carbohydrates were separated into six fractions: (1) organic acid (**OA**), (2) water-soluble carbohydrates (**WSC**) including sugars and fructans (Fraction **CA**), (3) starch (Fraction **CBl**), (4) neutral detergent soluble fiber (Fraction **CB2** = non-NDF-CA-CB1), (5) available NDF (Fraction **CB3** = NDF-NDICP-CC), and (6) unavailable NDF (Fraction **CC** = ADL × 2.4).

Based on the degradation rate in the rumen, feed protein was divided into non-protein nitrogen (**PA** = NPN), true protein (**PB**), and unavailable nitrogen (**PC** = ADICP). Furthermore, the true protein can be divided into three fractions: (1) the fast degradation part soluble in buffer solution (**PB1** = SCP-PA), (2) the medium degradation part insoluble in buffer solution but soluble in neutral detergent (**PB2** = CP-SCP-NDICP), and the slow degradation part insoluble in neutral detergent but soluble in acid detergent (**PB3** = NDICP-ADICP), as stated by Sniffen et al. (22).

### Statistical analysis

The collected data were subjected to the GLM procedure of SAS (SAS Institute Inc., Cary, NC, USA), considering the effects of raw material species and steam explosion processing along with their interaction as fixed factors in the model:


$$\begin{array}{l} {\text{Y}}_{\text{ijk}}\;=\text{ μ }+\;{\text{M}}_{\text{i}}\;+\;{\text{S}}_{\text{j}}\;+\text{ M}{\text{S}}_{\text{ij}}\;+\;{\text{e}}_{\text{ijk}} \end{array}$$

where Y_ijk_ represents every observation, μ is the general mean, M_i_ denotes the effect of raw material species (i = 1, 2, 3, 4, 5), S_j_ denotes the effect of the steam explosion processing (j = 0,1), MS_ij_ denotes the interaction of raw material species i with steam explosion processing j, and e_ijk_ is the random residual error. Duncan's test was used to compare the multiple means with differences declared significant at *P* < 0.05.

## Results

### Structural alteration after steam explosion processing

**Figure 1:**
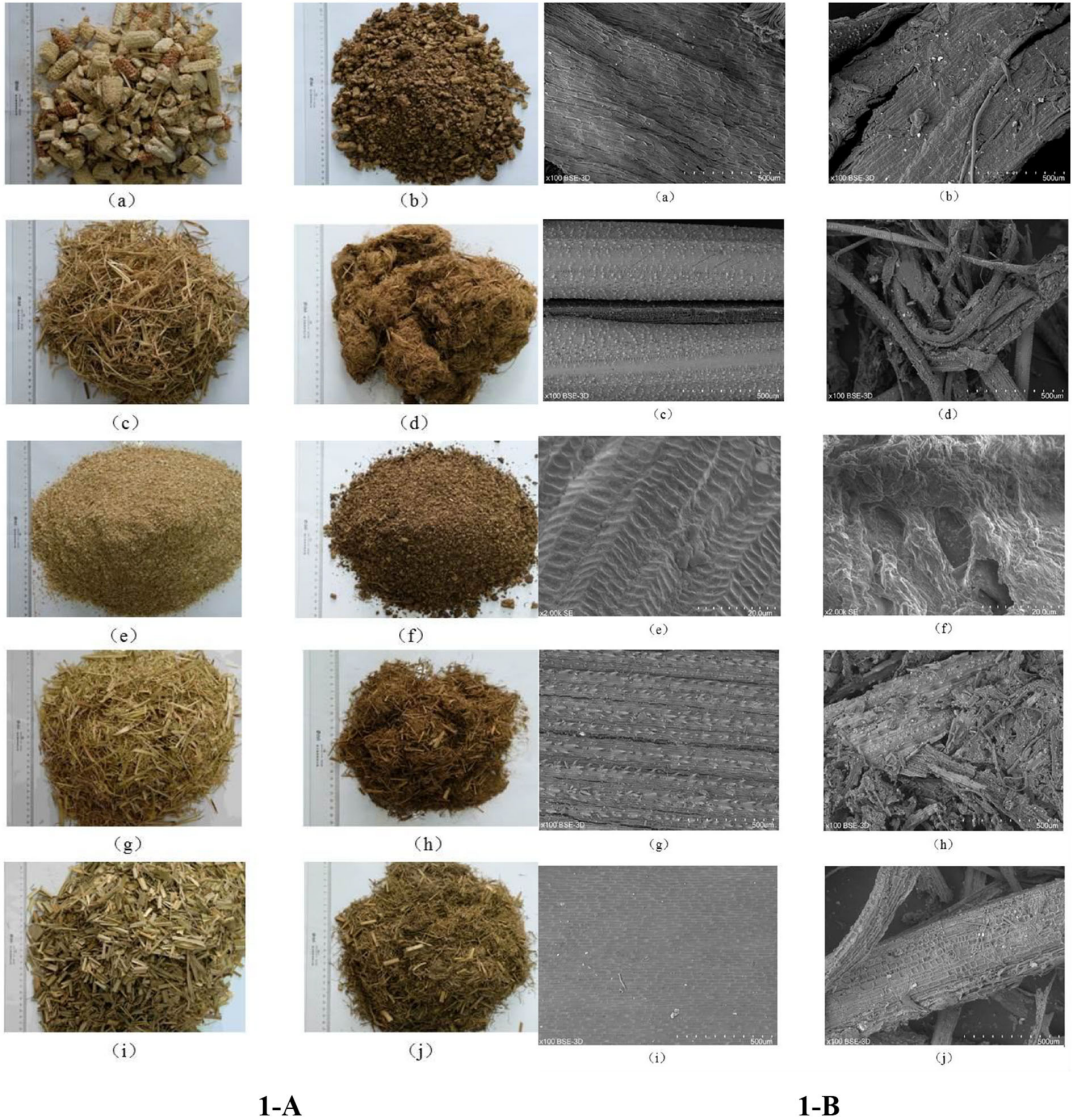
Physical appearance **(A)** and scanning electronic microscope observation **(B)** of feedstuffs before and after steam explosion processing (a: raw corn cob; b: steam-exploded corn cob; c: raw rice straw; d: steam-exploded rice straw; e: raw peanut shell; f: steamed-exploded peanut shell; g: raw millet stalk; h: steamed-exploded millet stalk; i: raw sugarcane tip; j: steam-exploded sugarcane tip).

As illustrated in Figure 1A, the appearance of corn cob, rice straw, peanut shell, millet stalk, and sugarcane tip changes obviously after steam explosion processing, wherein rice straw, millet stalk, and sugarcane tip became filamentary, while corn cob and peanut shell showed smaller mass and powder after steam explosion processing. Moreover, the color of all the feedstuff was somewhat dark after the steam explosion treatment. Furthermore, microstructural investigation (Figure 1B) by scanning electron microscopy (**SEM**) revealed that the surface of all the raw materials was intact, compact, and rigid with clear lines. In comparison, steam-exploded feedstuff showed a patchy, broken, irregular, and melted surface.

**Figure 2:**
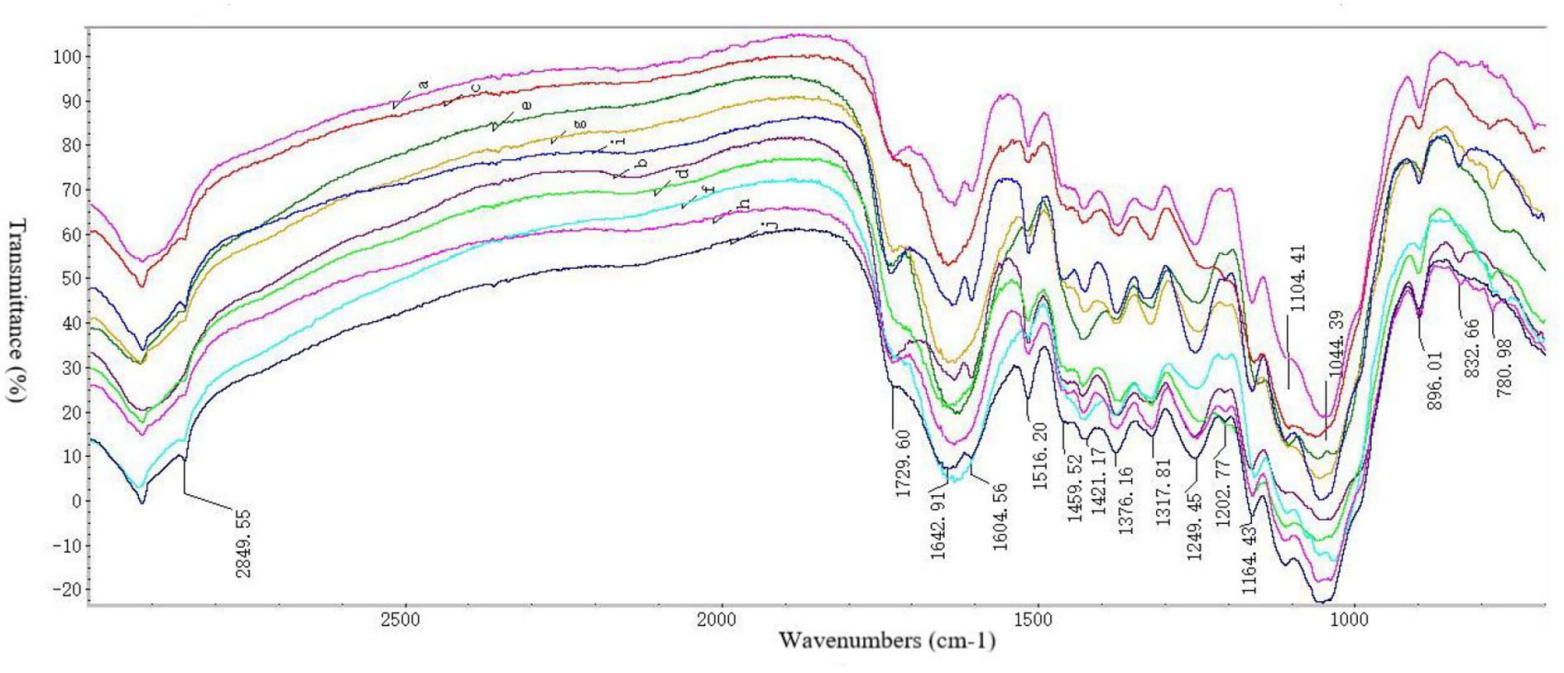
FTIR spectra of feedstuffs before and after steam explosion processing (a: raw corn cob; b: steam-exploded corn cob; c: raw rice straw; d: steam-exploded rice straw; e: raw peanut shell; f: steamed-exploded peanut shell; g: raw millet stalk; h: steamed-exploded millet stalk; i: raw sugarcane tip; j: steam-exploded sugarcane tip).

Furthermore, FTIR spectra of feedstuffs before and after processing are compared in Figure 2. In general, all the steam-exploded feedstuffs had almost the same peak distribution with corresponding raw materials. Obvious difference in the peak intensity was primarily found at 2,850, 1,730, 1,643, 1,605, 1,516, 1,460, 1,376, 1,318, 1,249, 1,164, 1,104, 896, 833, and 781 cm^−1^ among all the feedstuffs. After steam explosion processing, the peak intensity at 1,730, 1,249, and 781 cm^−1^ was decreased, and that at 1,605 and 1,104 was somewhat increased.

**Figure 3:**
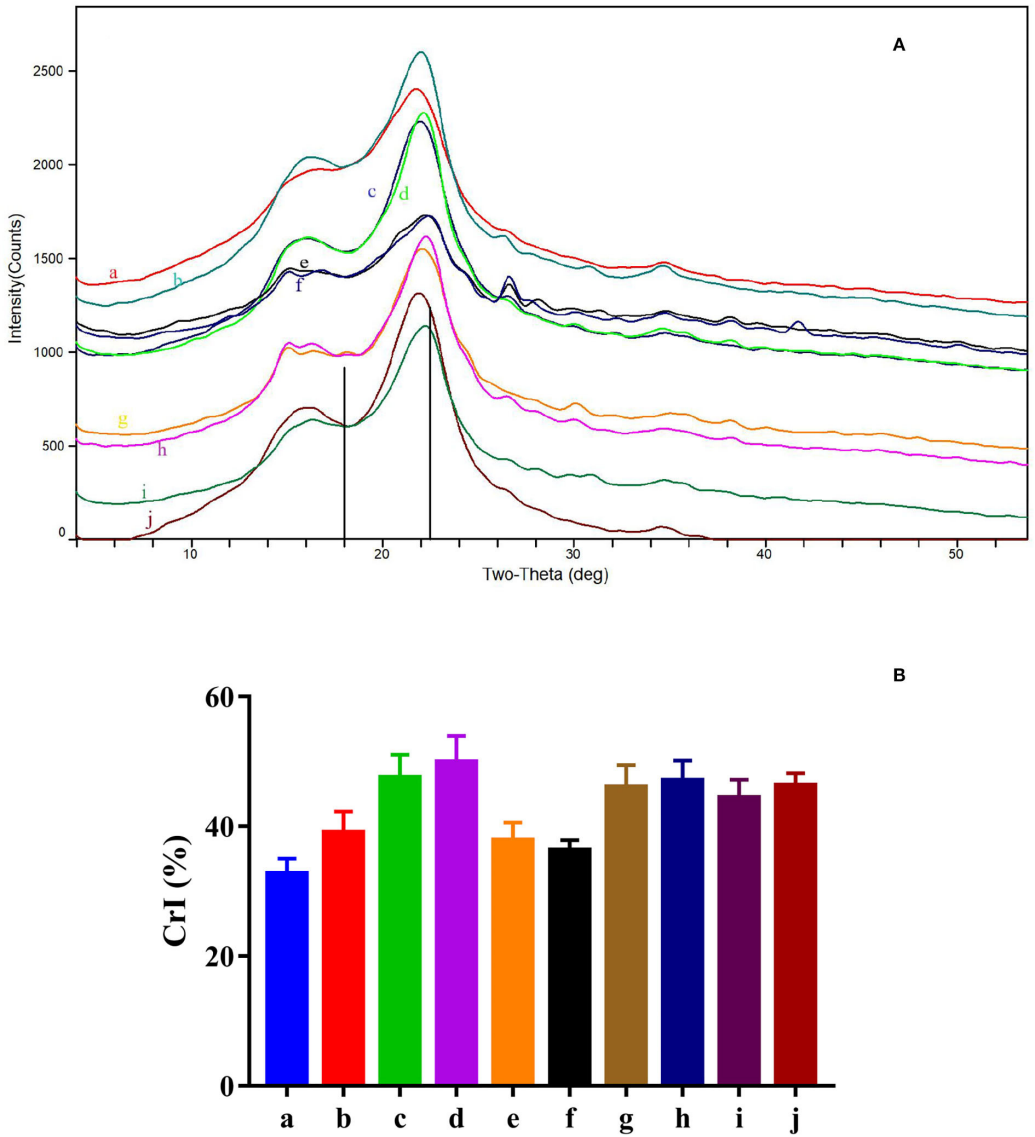
XRD images **(A)** and cellulose crystalline index (CrI) **(B)** of feedstuffs before and after steam explosion processing (a: raw corn cob; b: steam-exploded corn cob; c: raw rice straw; d: steam-exploded rice straw; e: raw peanut shell; f: steamed-exploded peanut shell; g: raw millet stalk; h: steamed-exploded millet stalk; i: raw sugarcane tip; j: steam-exploded sugarcane tip).

Diffraction images (Figure 3A) and cellulose crystalline index (Figure 3B) of feedstuffs before and after the steam explosion processing are shown in Figure 3. The comparison of the materials before and after steam explosion pretreatment revealed that the intensity of the main peak (2θ = 22.5°) was mostly intensified except for the peanut shell. Consistently, the estimated CrI of steam-exploded feedstuffs was numerically larger relative to that of the corresponding raw material.

### Variation in carbohydrate-protein fractions after steam explosion processing

The carbohydrate constituents of the feedstuffs before and after steam explosion processing are summarized in Table 1. The contents of NDF, ADF, ADL, and non-NDF carbohydrates of these byproducts varied in the ranges of 71.01–88.01, 42.00–60.45, 2.19–19.90, and 5.36–11.88%, respectively. Generally, all the carbohydrate constituents were significantly influenced (*P* < 0.01) by raw material species and steam explosion processing except that ADL content and CC proportion were not altered (*P* > 0.05) by steam explosion. Steam explosion processing led to the decrease (*P* < 0.01) of NDF, ADF, hemicellulose, and cellulose contents and the increase of non-NDF fraction and sugar concentration (except for peanut shell). Moreover, glucose, fructose, arabinose, and sucrose in sum accounted for over 97% of free sugars in the raw materials, where glucose and fructose were the two most sugars in the other four materials except that sucrose was in more concentration in the peanut shell. After steam explosion processing, the individual percentage of these sugars dramatically changed (*P* < 0.01). Based on the method of BCNRM in the analysis of carbohydrate fractions, fraction CB3 was decreased (*P* < 0.01), while fractions CA, CB1, and CB2 of the feedstuffs were increased (*P* < 0.01) by steam explosion processing.

**Table 1:** Carbohydrate constituents of feedstuff before and after steam explosion processing.

**Items^1, 2^**	**Raw material**					**Steam-exploded material**					**SEM**	***P*** **-value**		
	**Corn cob**	**Rice straw**	**Peanut shell**	**Millet stalk**	**Sugarcane tip**	**Corn cob**	**Rice straw**	**Peanut shell**	**Millet stalk**	**Sugarcane tip**		**M**	**S**	**MS**
NDF, %	88.01^a^	72.86^cd^	71.01^d^	79.06^b^	74.40^c^	64.15^f^	64.50^f^	57.09^g^	66.84^ef^	67.93^e^	1.00	< 0.01	< 0.01	< 0.01
ADF, %	44.86^bc^	45.60^bc^	60.45^a^	49.19^b^	42.00^cd^	42.13^cd^	47.86^b^	49.41^b^	47.87^b^	39.93^d^	1.52	< 0.01	< 0.01	< 0.01
ADL, %	4.87^f^	2.19^h^	19.90^a^	7.04^d^	6.43^de^	3.88^g^	4.10^fg^	17.07^b^	8.32^c^	6.06^e^	0.28	< 0.01	0.29	< 0.01
Hemi-cel, %	43.16^a^	27.26^bc^	10.56^cd^	29.88^b^	32.40^b^	22.02^bc^	16.65^c^	7.68^d^	18.97^c^	28.01^b^	2.79	< 0.01	< 0.01	< 0.01
Cellulose, %	39.98^b^	43.41^a^	40.55^ab^	42.15^a^	35.57^bc^	38.25^b^	43.76^a^	32.34^c^	39.55^b^	33.87^c^	1.58	< 0.01	< 0.01	< 0.01
Non-NDF, %	5.36^f^	5.50^f^	11.88^d^	7.39^e^	5.54^f^	29.60^a^	17.36^c^	17.81^c^	21.34^b^	11.42^d^	0.26	< 0.01	< 0.01	< 0.01
Total sugar, %	0.92^c^	1.96^bc^	9.52^a^	2.39^b^	1.32^bc^	3.06^b^	3.54^b^	7.47^ab^	3.05^b^	9.28^a^	1.27	< 0.01	< 0.01	< 0.01
Individual percentage, % total sugar														
Glucose	52.43^a^	49.60^b^	13.37^i^	45.29^c^	24.54^e^	15.30^h^	19.12^f^	24.54^e^	36.79^f^	16.52^g^	0.26	< 0.01	< 0.01	< 0.01
Fructose	33.24^c^	40.34^a^	16.44^g^	29.36^d^	34.82^b^	7.07^h^	7.21^h^	19.87^e^	18.03^f^	16.68^g^	0.15	< 0.01	< 0.01	< 0.01
Sucrose	8.40	7.13	67.83	19.18	23.10	2.65	ND	48.01	4.86	63.07	–	–	–	–
Arabinose	5.94^d^	1.66^h^	1.84^h^	3.15^f^	14.68^c^	54.33^a^	54.66^a^	4.78^d^	31.96^b^	2.31^g^	0.15	< 0.01	< 0.01	< 0.01
Galactose	ND	1.27	0.17	1.08	0.68	9.01	8.06	0.75	2.96	0.16	–	–	–	–
Xylose	ND	ND	0.38	0.72	ND	11.67	10.97	ND	5.41	ND	–	–	–	–
Mannose	ND	ND	ND	1.23	2.21	ND	ND	2.08	ND	1.27	–	–	–	–
Carbohydrate fractions of BCNRM, %														
CA (WSC)	0.98^f^	2.36^f^	10.63^a^	2.91^e^	1.58^g^	3.13^e^	3.89^d^	8.03^c^	3.89^d^	9.51^b^	0.17	< 0.01	< 0.01	< 0.01
CB1 (Starch)	0.52^de^	1.69^c^	0.64^d^	0.37^de^	0.34^e^	2.52^b^	4.62^a^	1.51^c^	2.28^b^	0.44^de^	0.09	< 0.01	< 0.01	< 0.01
CB2	3.87^d^	1.45^e^	0.59^f^	4.11^d^	3.62^d^	23.95^a^	8.85^c^	8.27^c^	15.17^b^	1.47^e^	0.18	< 0.01	< 0.01	< 0.01
CB3	74.30^a^	64.26^b^	18.85^h^	60.24^c^	55.48^d^	53.51^e^	52.90^e^	11.72^i^	45.18^g^	49.71^f^	0.47	< 0.01	< 0.01	< 0.01
CC	11.10^f^	5.25^h^	47.75^a^	16.89^d^	15.42^de^	9.31^g^	9.84^fg^	40.97^b^	19.98^c^	14.54^e^	0.68	< 0.01	0.29	< 0.01

^1^ NDF, neutral detergent fiber; ADF, acid detergent fiber; ADL, acid detergent lignin; Hemi-cel, hemicellulose; BCNRM, beef cattle nutrient requirement model (2016); non-NDF = 100-(CP+NDF+Fat+Ash), WSC, water soluble carbohydrate, Fraction CA = WSC; Fraction CBl = starch; Fraction CB2 = non-NDF-CA-CB1; Fraction CB3 = NDF-NDICP-CC; Fraction CC = ADL × 2.4.

^2^ M, the effect of raw material; S, the effect of processing; MS, the interaction of raw material by processing; SEM, standard error of means; ND, not detected; ^a−*h*^ Values with different superscripts in the same row denote difference at P < 0.05.

The protein fractions of these byproducts before and after steam explosion processing are presented in Table 2. These byproducts contained a relatively low content of crude protein (3.52–9.37%), where NPN, SCP, NDICP, and ADICP varied (*P* < 0.01) in the range of 0.60–3.35, 1.13–3.48%, 1.93–3.50%, and 0.74–2.48%, respectively. After steam explosion processing, the contents of these protein constituents were altered (*P* < 0.01), in which CP content and fraction PB3 were decreased while NPN and ADICP were increased in all five materials, along with inconsistent changes in SCP and NDICP.

**Table 2:** Protein constituents of feedstuff before and after steam explosion processing.

**Items^1, 2^**	**Raw material**					**Steam-exploded material**					**SEM**	***P*** **-value**		
	**Corn cob**	**Rice straw**	**Peanut shell**	**Millet stalk**	**Sugarcane tip**	**Corn cob**	**Rice straw**	**Peanut shell**	**Millet stalk**	**Sugarcane tip**		**M**	**S**	**MS**
CP, %	3.52^e^	5.79^c^	8.97^a^	4.75^d^	9.37^a^	2.94^e^	4.25^d^	8.28^b^	4.30^d^	8.97^a^	0.21	< 0.01	< 0.01	< 0.01
SCP, %	1.85^b^	1.13^c^	3.28^ab^	1.62^bc^	3.48^ab^	1.07^c^	1.46^bc^	4.28^a^	1.89^b^	3.68^ab^	0.40	< 0.01	< 0.01	< 0.01
NDICP, %	2.02^b^	3.36^a^	3.59^a^	1.93^b^	3.50^a^	1.34^c^	1.97^b^	3.40^a^	1.68^bc^	3.69^a^	0.17	< 0.01	0.03	< 0.01
PA (NPN, %)	0.60^f^	0.98^d^	3.10^b^	1.65^c^	3.35^b^	0.64^f^	1.35^cd^	3.31^b^	1.75^c^	3.69^a^	0.10	< 0.01	< 0.01	< 0.01
PB1, %	1.25^a^	0.21^cde^	0.31^cd^	0.03^e^	0.08d^e^	0.43^c^	0.11^de^	0.97^b^	0.13^de^	0.06d^e^	0.08	< 0.01	0.55	< 0.01
PB2, %	0.10^f^	1.30^d^	2.10^b^	1.48^cd^	2.40^a^	0.54^fg^	0.83^e^	0.60^e^	0.73^e^	1.61^c^	0.19	< 0.01	0.90	< 0.01
PB3, %	1.22^c^	2.39^a^	1.12^c^	1.17^c^	2.33^a^	0.31^d^	0.33^d^	0.17^d^	0.20^d^	1.57^b^	0.08	< 0.01	< 0.01	< 0.01
PC (ADICP, %)	0.81^f^	0.98^f^	2.48^b^	0.74^f^	1.18^edf^	1.03^ef^	1.64^cd^	3.24^a^	1.50^de^	2.12^bc^	0.16	< 0.01	< 0.01	< 0.01

^1^ CP, crude protein; SCP, soluble crude protein; NDICP, neutral detergent insoluble crude protein; ADICP, acid detergent insoluble crude protein; NPN, non-protein nitrogen; PA = NPN, PB1 = SCP-PA, PB2 = CP-SCP-NDICP, PB3 = NDICP-ADICP, PC = ADICP as stated by Sniffen et al. (22).

^2^ M, the effect of raw material; S, the effect of processing; MS, the interaction of raw material by processing; SEM, standard error of means; ^a−*g*^ Values with different superscripts in the same row denote difference at P < 0.05.

### *In vitro* rumen fermentation profile before and after steam explosion processing

**Figure 4:**
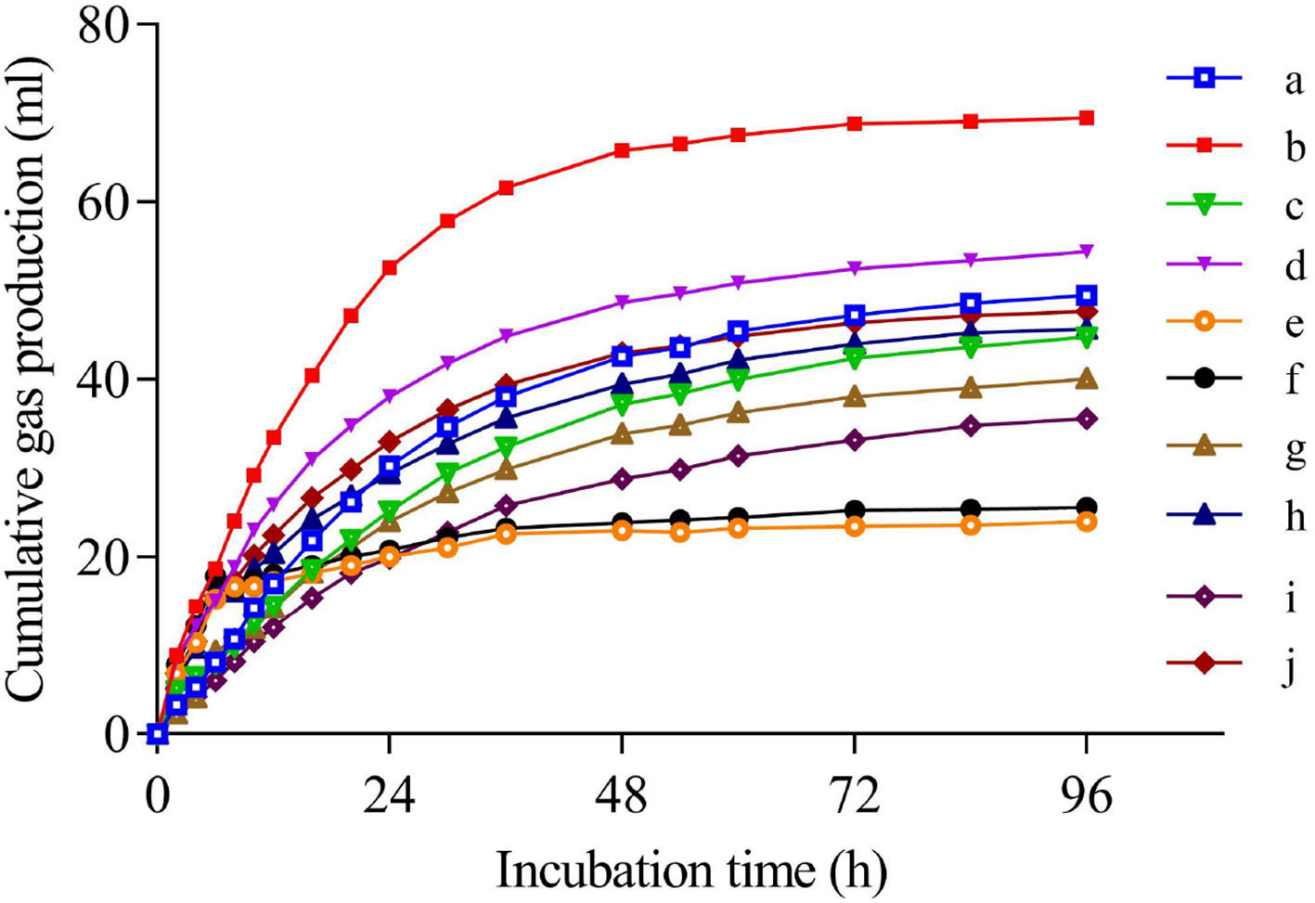
*In vitro* gas production dynamics of feedstuff before and after steam explosion processing (a: raw corn cob; b: steam-exploded corn cob; c: raw rice straw; d: steam-exploded rice straw; e: raw peanut shell; f: steamed-exploded peanut shell; g: raw millet stalk; h: steamed-exploded millet stalk; i: raw sugarcane tip; j: steam-exploded sugarcane tip).

The dynamic curves of cumulative gas production are illuminated in Figure 4. In general, all the curves almost reached a plateau after 96 h of incubation. There were apparent gaps among the curves of different feedstuffs, and the gaps between the curves of each material before and after steam explosion processing were also obvious except for the peanut shell. Statistically, gas production parameters, including GP_24_, GP_96_, b, and c, as well as pH and NH_3_-N concentration, were affected (*P* < 0.01) by both raw material species and steam explosion processing, where steam-exploded corn cob produced the most gas production and that of peanut shell was the least (Table 3). In detail, after steam explosion processing, the gas production rate and gas production volume of these byproducts remarkably increased (*P* < 0.01), total VFA concentration tended to increase (*P* = 0.05), and fluid pH value and NH_3_-N concentration declined (*P* < 0.01) for most of the feedstuff. Furthermore, the individual proportion of all the VFAs except for isovalerate varied (*P* < 0.01) in the different materials, and the individual proportions of propionate and isobutyrate were increased (*P* < 0.01) by steam explosion processing.

**Table 3:** *In vitro* rumen fermentation profile of feedstuff before and after steam explosion processing.

**Items^1, 2^**	**Raw material**					**Steam-exploded material**					**SEM**	***P*** **-value**		
	**Corn cob**	**Rice straw**	**Peanut shell**	**Millet stalk**	**Sugarcane tip**	**Corn cob**	**Rice straw**	**Peanut shell**	**Millet stalk**	**Sugarcane tip**		**M**	**S**	**MS**
GP_24_, ml	30.3^cd^	25.2^d^	20.1^e^	24.0^d^	19.9^e^	52.6^a^	38.1^b^	20.8^e^	29.4^cd^	33.0^c^	1.5	< 0.01	< 0.01	< 0.01
GP_96_, ml	49.5^c^	44.8^d^	24.0^g^	40.1^e^	35.6^f^	70.2^a^	54.5^b^	25.6^g^	45.7^d^	47.8^cd^	1.4	< 0.01	< 0.01	< 0.01
b, ml	51.2^b^	47.0^c^	22.5^f^	41.1^d^	36.9^e^	70.5^a^	53.3^b^	23.6^f^	44.4^cd^	47.0^c^	1.1	< 0.01	< 0.01	< 0.01
c, %/h	3.7^d^	3.2^d^	14.4^b^	3.6^d^	3.3^d^	5.5^c^	5.4^c^	15.8^a^	5.1^c^	5.4^c^	0.2	< 0.01	< 0.01	0.15
pH	6.69^c^	6.85^a^	6.91^a^	6.82^ab^	6.81^ab^	6.54^d^	6.65^cd^	6.93^a^	6.73^bc^	6.85^a^	0.04	< 0.01	< 0.01	0.02
NH_3_-N, mg/dl	32.55^ab^	32.26^ab^	33.61^ab^	34.47^ab^	35.44^a^	27.30^c^	26.99^c^	31.38^b^	31.16^b^	33.65^ab^	1.14	< 0.01	< 0.01	0.41
TVFA, mM	37.39^ab^	30.79^ab^	29.30^b^	36.46^ab^	30.16^b^	37.38^ab^	34.63^ab^	34.64a^b^	36.33^ab^	38.89^a^	2.72	0.25	0.05	0.46
Individual VFA proportion, % TVFA				
Acetate	61.37^a^	59.72^ab^	57.36^c^	61.37^a^	59.57^ab^	59.98^ab^	60.89^a^	58.36^bc^	61.22^a^	61.61^a^	0.68	< 0.01	0.23	0.15
Propionate	15.65^a^	15.39^ab^	12.77^d^	14.40^c^	15.04^b^	15.68^a^	15.44^a^	12.99^d^	15.21^ab^	15.41^ab^	0.18	< 0.01	0.02	0.23
Ace:Prop	3.92^cde^	3.88^de^	4.50^a^	4.26^b^	3.96^cd^	3.83^e^	3.94^cde^	4.49^a^	4.03^c^	4.00^cd^	0.04	< 0.01	0.07	0.01
Butyrate	1.84^b^	2.25^b^	3.48^a^	2.57^b^	2.24^b^	2.17^b^	2.13^b^	3.59^a^	2.12^b^	2.23^b^	0.27	< 0.01	0.87	0.69
Isobutyrate	7.75^b^	7.08^c^	7.50^b^	7.01^c^	6.82^c^	9.03^a^	7.60^b^	7.62^b^	7.68^b^	7.63^b^	0.20	< 0.01	< 0.01	0.10
Valerate	2.53^bc^	2.66^b^	3.25^a^	2.70^b^	2.85^b^	2.62^b^	2.26^c^	3.31^a^	2.52^bc^	2.62^b^	0.10	< 0.01	0.05	0.12
Isovalerate	0.19	0.15	0.25	0.18	0.16	0.24	0.15	0.31	0.16	0.23	0.05	0.14	0.33	0.86

^1^ GP_24_, cumulative gas production after 24 h of *in vitro* rumen incubation; b, asymptotic gas production; c, the rate of gas production; NH_3_-N, ammonia-nitrogen; TVFA, total volatile fatty acids; Ace:Prop, the ratio of acetate and propionate.

^2^ M, the effect of raw material; S, the effect of processing; MS, the interaction of raw material by processing; SEM, standard error of means; ^a−*g*^ Values with different superscripts in the same row denote difference at P < 0.05.

**Figure 5:**
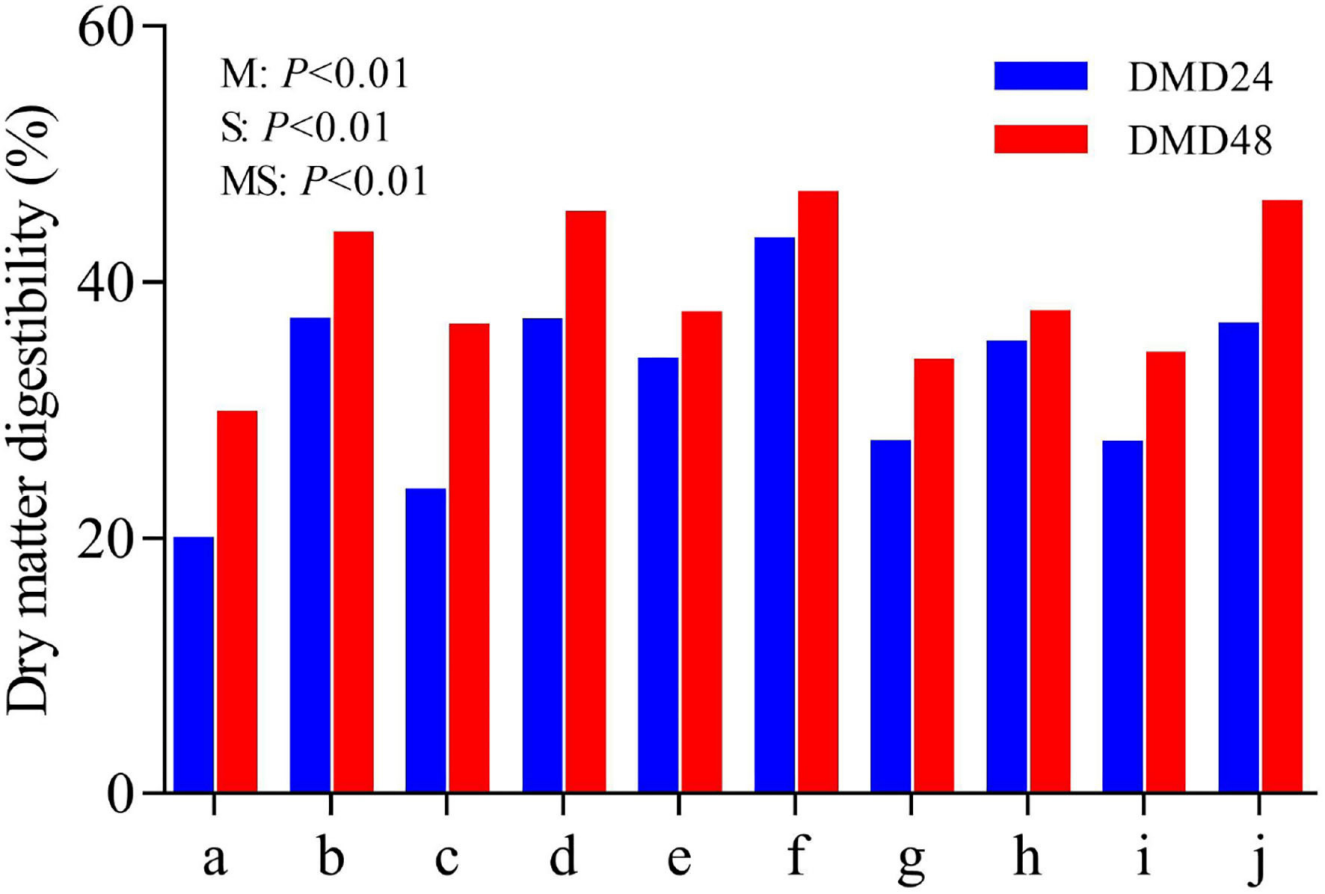
*In vitro* dry matter digestibility (DMD) of feedstuff before and after steam explosion processing (a: raw corn cob; b: steam-exploded corn cob; c: raw rice straw; d: steam-exploded rice straw; e: raw peanut shell; f: steamed-exploded peanut shell; g: raw millet stalk; h: steamed-exploded millet stalk; i: raw sugarcane tip; j: steam-exploded sugarcane tip; M: the effect of raw material; S: the effect of processing; MS: the interaction of raw material by processing).

The *in vitro* rumen digestibility of DM (DMD) at 24 and 48 h of incubation is illustrated in Figure 5. The DMD at 24 and 48 h of these byproducts varied (*P* < 0.01) in the range of 19.67–33.67 and 29.54–37.29%, and were increased (*P* < 0.01) up to 35.00–43.07 and 37.39–46.66% by steam explosion processing, respectively.

**Figure 6:**
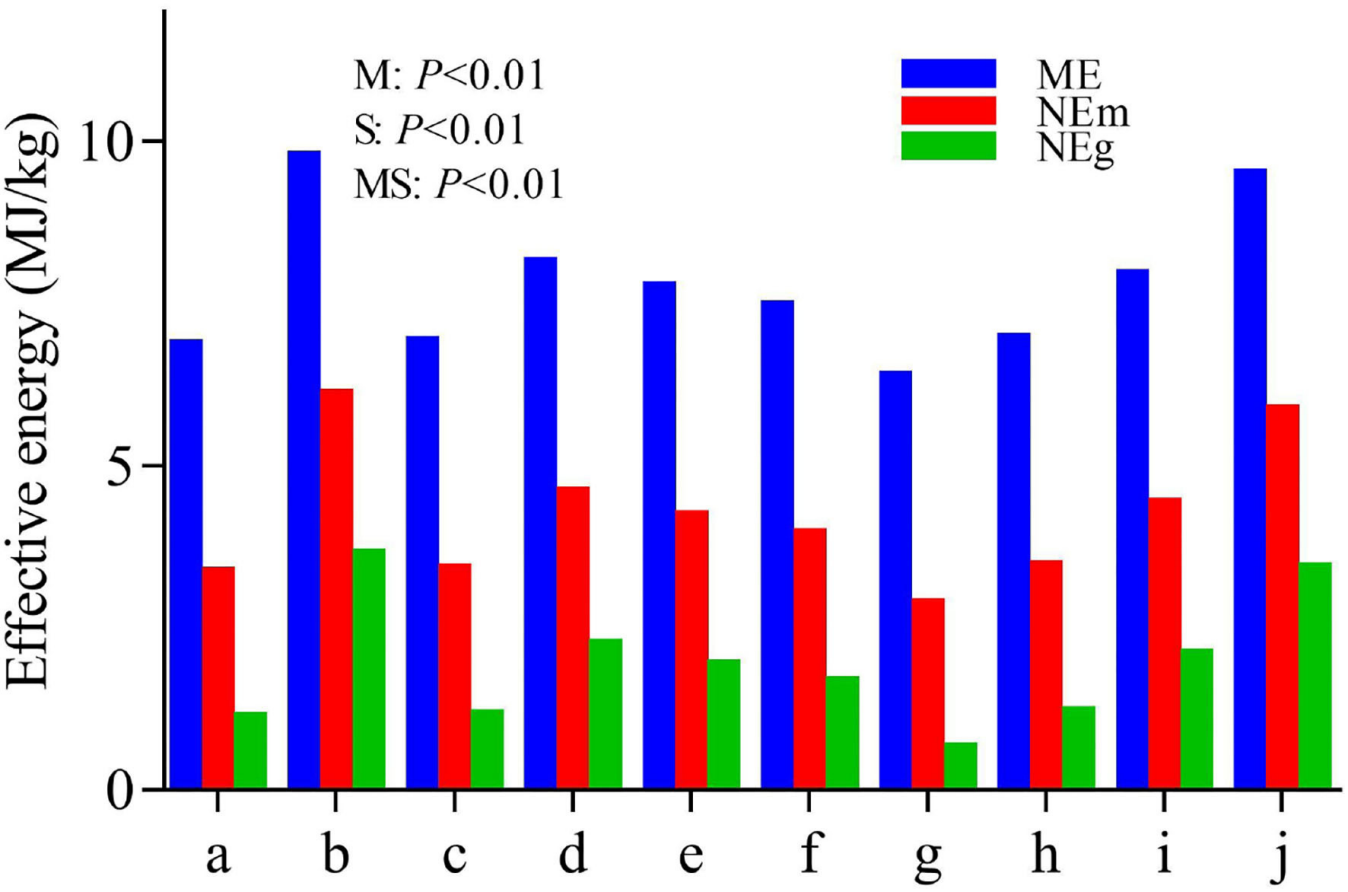
Predicted effective energy of feedstuff before and after steam explosion processing (a: raw corn cob; b: steam-exploded corn cob; c: raw rice straw; d: steam-exploded rice straw; e: raw peanut shell; f: steamed-exploded peanut shell; g: raw millet stalk; h: steamed-exploded millet stalk; i: raw sugarcane tip; j: steam-exploded sugarcane tip; M: the effect of raw material; S: the effect of processing; MS: the interaction of raw material by processing).

The predicted effective energy values of these byproducts are shown in Figure 6. The predicted energy values of these byproducts except for peanut shells were somewhat increased by steam explosion processing. The values of ME, NEm, and NEg in the raw materials were in the range of 6.38–7.94, 2.87–4.42, and 0.64–2.10 MJ/kg, respectively, which were increased to 6.96–9.76, 3.46–6.10, and 1.20–3.64 MJ/kg, respectively, after steam explosion processing.

## Discussion

### Structure comparison before and after steam explosion

After steam explosion processing, the appearance of the physical structure of the feedstuff was obviously altered in the present study. The darker color was mainly owed to the browning reaction under the hygrothermal environment of steam explosion. The stronger the steam explosion intensity (log R_0_; R_0_ = t × e^(*T*−100)/14.75^) (23), the darker the color of steam-exploded feedstuff. With different physicochemical structures of raw materials, rice straw, millet stalk, and sugarcane tip became filamentary structures, while corn cob and peanut shell showed smaller mass and powder after the steam explosion. Such changes in appearance would likely promote feed ingestion and digestion for ruminants. Moreover, surface morphology is a critical factor influencing feed digestibility because it would affect the contact area of enzymes or bacteria to the biomass, and surface adsorption is the first step of microbial digestion. Steam explosion rendered the morphology to become patchy, broken, irregular, and melted, which subsequently would likely increase the specific surface area of the feedstuff. Such alterations in the structure might be interpreted as some components like hemicellulose are hydrolyzed or melted on the surface when the materials are cooked at high pressures and high temperatures, and the softened materials are torn out by the shear force of water steam when high pressure is sharply released at explosion (6). The decrease in FTIR peak intensity at 1,730 cm^−1^, representing the carbonyl stretching, can be attributed to the acetyl, glucuronic acid, and ferulic ester groups of polysaccharides (24, 25), which indicated that steam explosion processing resulted in serious cleavage of acetyl groups. Consistently, the band at 1,249 cm^−1^, originating from –CH_2_ bending vibrations of hemicellulose or aromatic ring vibration of lignin (24, 26), was also weakened. Meanwhile, the characteristic peak for lignin at 1,605 cm^−1^ and the band at 1,104 cm^−1^ indicating the vibration of arabinosyl side chains was intensified (24), indicating that the structures of lignin and hemicellulose did alter during processing. Additionally, differences in peak intensity at 2,850, 1,643, 1,516, 1,460, 1,376, 1,318, 1,164, 896, 833, and 781 cm^−1^ among all the materials should be mainly owed to the different components and properties. Furthermore, XRD analysis also revealed that the CrI of most feedstuffs was increased by steam explosion processing, which might be due to the increased relative content of cellulose, as well as recrystallization (7, 10, 27). It is documented that crystallinity is an important characteristic of lignocelluloses for hydrolysis/digestibility but is not the sole effective factor (28). The properties of raw material, as well as processing conditions like pressure, temperature, and residence time, could influence the effectiveness of steam explosion processing (6, 7), thus various responses were found in the structure of different byproducts. In a word, the alterations in the morphological structure and functional groups indicate that these byproducts might become easier to digest after steam explosion processing.

### Carbohydrate-protein fractions before and after steam explosion

Carbohydrates are the major source of energy for animals. Various constituents contribute to the overall carbohydrate content in the diet, each with unique chemistry and availability. Based on the differences in chemistry and functionality, carbohydrates can be broadly partitioned into non-neutral detergent fiber (non-NDF) carbohydrates and neutral detergent fiber (NDF) carbohydrates. Referring to the Nutrient Requirements of Beef Cattle (21), dietary carbohydrates are further separated into six fractions mainly based on analytical methodology, similarity in digestion rates, and availability of the fractions in the rumen. In this study, analyzing individual fractions of carbohydrates would help us to make clear specific changes during steam explosion processing, better predicting the digestion potential and functionality of the feedstuff.

In the present study, the contents of carbohydrate constituents and carbohydrate fractions varied much in these byproducts and were remarkably altered by steam explosion processing. In comparison, non-NDF fraction mainly includes OA, WSC, and soluble fiber, and is typically more digestible than NDF fraction which is composed of hemicellulose, cellulose, and lignin (21). Such a high NDF content and a low non-NDF fraction of these byproducts indicate that they are less digestible and low in nutritional value. Much research shows that steam explosion processing could result in remarkable physicochemical changes in various kinds of biomass (7, 29). The FTIR absorption peak of the acetyl group (–C=O), which is a characteristic peak of hemicellulose, is remarkably decreased in different kinds of materials after steam explosion pretreatment (5, 7). Consistently, steam explosion processing led to a decrease in NDF, ADF, hemicellulose, and cellulose contents and an increase in non-NDF fraction and sugar concentration. The hydrolysis of hemicellulose (by 13.55–48.98%) and cellulose (by 0–20.25%) primarily accounts for the variation of these constituents, where hemicellulose is more susceptible to different kinds of treatments. Chang et al. (30) report that cellulose, hemicellulose, and lignin contents of corn stover are decreased by 8.47, 50.45, and 36.65%, respectively, after steam explosion processing. He et al. (7) and Nie et al. (31) report that more than 70% of hemicellulose in *Hippophae rhamnoides* and corn stover is converted during steam explosion processing. At the same time, the breakage or depolymerization of NDF fraction generating OA, WSC, and other small molecule compounds would increase the fraction of non-NDF (by 49.92–452.24%). Furthermore, the free sugar content was increased (by 27.62–603.03%) in most of the feedstuff, and their sugar profiles were remarkably altered. Moreover, as the common monomers of hemicellulose, the increased percentages of arabinose, galactose, and xylose coincide with the decrease of hemicellulose. The above-mentioned findings indicate that the carbohydrate constituents of these byproducts were dramatically altered by steam explosion processing. Meanwhile, raw materials with various properties would show different responses to steam explosion processing. The effectiveness of steam explosion processing could be affected by the properties of raw material, as well as the pressure, temperature, and residence time of processing (6, 7). As a consequence, based on the method of BCNRM in the analysis of carbohydrate fractions, fraction CB3 was decreased, while fractions CA, CB1, and CB2 were increased, indicating that these byproducts become more easily digestible given that the digestion rates of WSC, starch, and soluble fiber are usually faster than that of the available NDF (21).

Based on the degradation rate in the rumen, feed protein is divided into non-protein nitrogen, true protein, and unavailable nitrogen, where the true protein can be further divided into three fractions (22). Even though there is usually a low crude protein content (3.52–9.37%) in crop byproducts, they still act as an important protein source because such roughage usually accounts for a large part of ruminant diets. After steam explosion processing, CP and fraction PB3 were decreased, while NPN and ADICP were increased along with the inconsistent changes in SCP and NDICP. Such variations could be interpreted based on the fact that protein degradation and Maillard reaction occur under the severe conditions of steam explosion, resulting in the increase of NPN and ADICP (32), while the loss of nitrogen nutrient and measurement error might account for the decrease of CP content. Given the differences in the digestion rate and availability in the rumen, i.e., ADICP is usually considered as the unavailable protein, and NPN has a lower utilization efficiency relative to true protein in ruminant nutrition (15); such alterations would likely lower the nutritional value of feed protein. Thus, it is suggested that steam explosion processing would likely discount the nutritional value of protein in lignocellulosic biomass, which is noteworthy when formulating ruminant diets.

### *In vitro* rumen fermentation profile before and after steam explosion

Due to its high correlation with *in vivo* study and easy operation, *in vitro* rumen incubation becomes a common method to evaluate the fermentation profile of feedstuff, such as gas production, nutrient digestibility, and even effective energy content. As microbial fermentation of feed samples in the rumen would release gases (CO_2_, CH_4_, H_2_, and H_2_S), which are the metabolites of microorganisms under certain metabolic pathways, the volume or speed of gas production can be used to reflect the degree and speed of nutrient fermentation (16). Gas production is mainly related to the chemical composition of the feedstuff, being that a more digestible substrate would result in more gas volume. In the present study, the gas production rate and gas production volume of these byproducts were remarkably increased after steam explosion processing, in line with the deduction of the aforementioned physicochemical changes. Consistently, the content of total VFA, the main product of carbohydrate fermentation in the rumen, tended to increase, and the pH value and NH_3_-N concentration decreased. It is suggested that more substrate is fermented, generating more gas production, VFA, and microbial protein, finally resulting in the decrease of pH value and NH_3_-N concentration. Furthermore, the individual proportions of propionate and isobutyrate were increased. In general, VFA is the main substrate of energy supply for ruminants. The metabolic pathway of propionate generation is more efficient in energy transformation relative to those of acetate and butyrate production in rumen given that there is less energy loss of gas emission (33). Thus, it is suggested that steam-exploded feedstuff becomes more efficient in energy supply for ruminants relative to the raw materials. Isoacids like isobutyrate and isovalerate primarily derive from the metabolites of amino acids valine, leucine, isoleucine, and proline (34). The increase in isobutyrate levels implies that more protein is fermented by the oxidative pathway of decarboxylation or deamination, i.e., more protein is available in the steam-exploded feedstuff. As mentioned above, steam explosion processing might make the nutrients become widely exposed to microbes and become more easily digested. Consistently, all the steam-exploded byproducts had a higher DM digestibility (DMD at 24 h and 48 h of incubation) relative to their raw materials. Many studies have proven that nutrient digestibility is one of the most important factors influencing the forage intake of ruminants. The increase in DMD might contribute to the increase in feed intake.

According to the study by Menke and Steingass (20), gas production after 24 h of *in vitro* rumen incubation is highly related to the metabolizable energy content of the ruminant feed. Thus, *in vitro* rumen gas production is often used to predict the available energy value of the ruminant feed. In the present study, the ME, NEm, and NEg values of these byproducts (except for peanut shells) were much increased after steam explosion processing, coinciding with the alterations in their carbohydrate constituents. In comparison, the high lignin content and low hemicellulose content might primarily account for the less improvement in peanut shells. Noteworthy, it is suggested that there exists a material-specific response to steam explosion processing. Additionally, more comprehensive experiments are required to further confirm the effectiveness of steam explosion processing on the utilization of crop byproducts.

## Conclusion

The results showed that these five crop byproducts possessed different physicochemical properties and rumen fermentation profiles, most of which were improved by steam explosion processing, i.e., more rough morphological surface, much-altered chemical structure, more digestible carbohydrate fractions, faster gas production rate, higher dry matter digestibility, and more available energy. Noteworthy, not all the materials could expect an improved result, especially those with high lignin content and low hemicellulose content. It is suggested that steam explosion processing could intensify the nutritional values of most crop byproducts, with different responses of raw materials and caution of protein heat damage.

## Data availability statement

The original contributions presented in the study are included in the article/supplementary material, further inquiries can be directed to the corresponding author/s.

## Author contributions

LH: conceptualization, methodology, writing, and reviewing and editing. YH: resources, methodology, investigation, and draft. LS: methodology, investigation, data collection, and formal analysis. ZZ: validation and supervision. HW: conceptualization, methodology, visualization, and supervision. All authors contributed to the article and approved the submitted version.

## Funding

This work was financially supported by the National Natural Science Foundation of China (Grant No. 32102562) and the China Agriculture Research System of MOF and MARA (CARS-39; CARS-37).

## Conflict of interest

The authors declare that the research was conducted in the absence of any commercial or financial relationships that could be construed as a potential conflict of interest.

## Publisher's note

All claims expressed in this article are solely those of the authors and do not necessarily represent those of their affiliated organizations, or those of the publisher, the editors and the reviewers. Any product that may be evaluated in this article, or claim that may be made by its manufacturer, is not guaranteed or endorsed by the publisher.

## Footnotes

^1^FAOSTAT (2022). Available online at: http://www.fao.org/faostat/zh/#data/QC.
